# Supplementary material for: Quantum interferometric generation of polarization entangled photons
Source: Sci Rep. 2018 Oct 24;8:15733. doi: 10.1038/s41598-018-33876-z (PMC6200815; doi:10.1038/s41598-018-33876-z)
Supplement: Supplementary file 1 — Supplementary Information [file 41598_2018_33876_MOESM1_ESM.pdf]

Supplementary Information

Quantum interferometric generation of polarization entangled photons

Haruka Terashima, Satoshi Kobayashi, Takaho Tsubakiyama,

and Kaoru Sanaka [a)]

Department of Physics, Tokyo University of Science, Shinjuku-ku, Tokyo 162-8601, Japan,

Electronic mail: [a] sanaka@rs.tus.ac.jp

Supplementary Note 1

In our scheme, we need to tilt the HWP1 as shown in Fig. 1(a) to adjust the relative phase between the photon pairs from the first and second SPDCs. Supplementary Fig. S1 shows the top and side view of HWP1. When the HWP1 is set with no tilting angle, the heights of the clockwise (CW) and counterclockwise (CCW) beam are same. When the HWP1 is set with a tilting angle, the heights of the CW and CCW beam become different. The visibility of interference fringes deteriorates because the height difference causes spatial mode mismatch on the output of the Sagnac interferometer.

The visibility of interference fringes is expected to be higher when using a singlemode fiber (S.M.F.) to collect the beam output from the interferometer than when using a multimode fiber (M.M.F) because the S.M.F is filtering out the spatial mode-overlapping area of the CW and CCW beams.

Supplementary Note 2

When we use singlemode fibers for collecting the output beams from the interferometer shown in Fig. 1(a), it is possible to filtering out the spatial mode-overlapping area of the CW and CCW beams if the heights of these beams are slightly different. We performed an additional experiment using a setup as shown in Supplementary Fig. S1 to observe the difference of interference visibilities between a multimode fiber (M.M.F.) and a singlemode fiber (S.M.F.) to collect the output beams from the Sagnac interferometer.

Supplementary Fig. S2(a) to (c) show the plot of laser intensity output from the interferometer when the tilting angle of HWP1 is set at 2° as a function of the angles of HWP for laser using the M.M.F. only, and the M.M.F. with 1 mm diameter irises, and the S.M.F. only. We estimated the visibilities by fitting a theoretical function to these plot data. Supplementary Fig. S2(d) shows the plot of measured visibilities as a function of the tilting angles of HWP1. The visibilities with the M.M.F. deteriorate rapidly depending on the tilting angles (square dots) due to low spatial mode overlapping. The visibilities with the M.M.F. and irises deteriorate moderately (triangle dots) owing to a spatial mode filtering effect. On the other hand, the visibilities with the S.M.F. is almost constant against for the angles (circle dots) owing to a single spatial mode filtering effect. The results clearly show that the effect of S.M.F. coupling is very useful to recover the visibilities against for HWP1 tilting angle.

An 808 nm wavelength diode laser is used as the light source for this experiment because the laser beam is very efficient to measure the visibilities. Similarly, when using the S.M.F to collect the polarization-entangled photons generated by our scheme, improvement of the visibilities and fidelities can be expected.

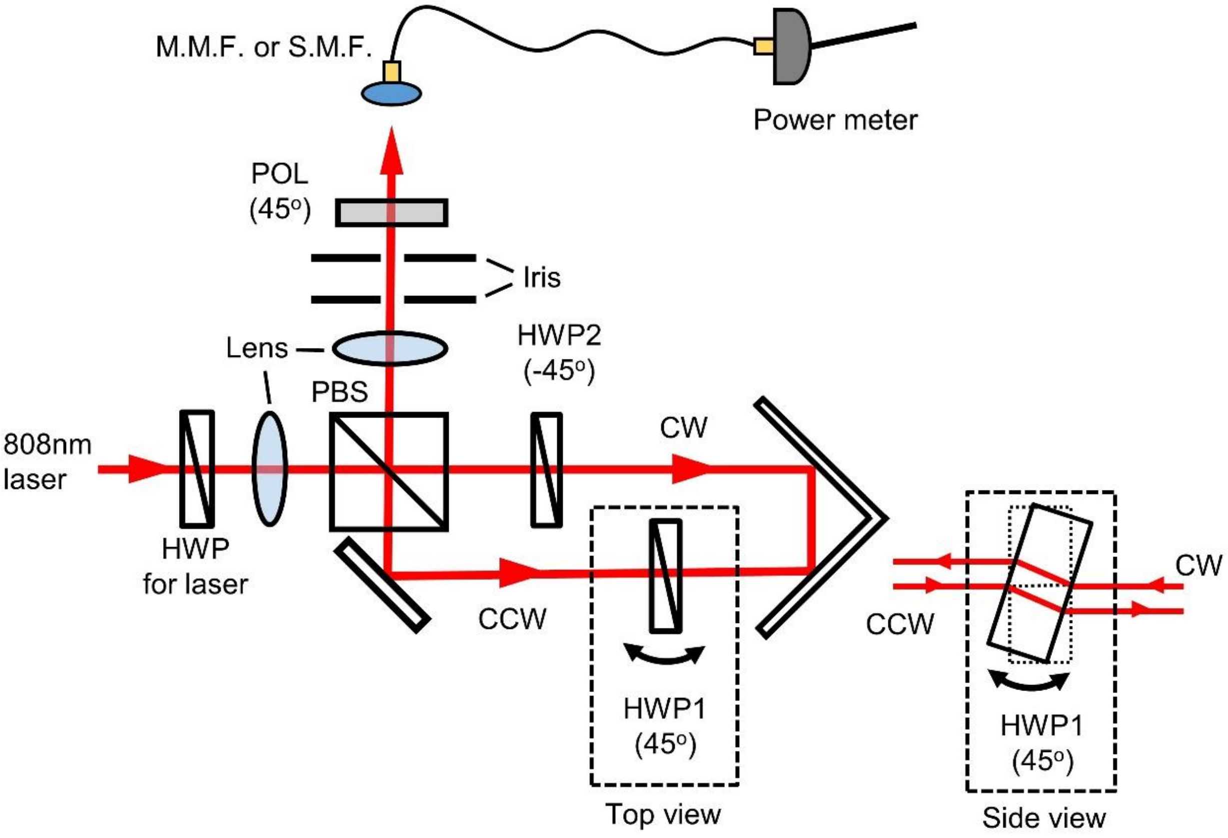

Supplementary Figure S1. Experimental setup to measure interference visibilities depending on spatial mode overlapping.

An 808 nm wavelength diode laser is used as the input light source for the Sagnac interferometer that has been used for generating polarization entangled photons as shown in Fig.2. The focus and collimation conditions are similar to those in the setup in Fig.2. The polarization state of the laser is set to horizontal initially and converted into arbitrary linear polarization states using a half wave plate (HWP) for laser. The horizontal component of the laser passes through the polarizing beam splitter (PBS) and round trips the setup in the clockwise (CW) direction. The vertical component of the laser round trips the setup in the counterclockwise (CCW) direction. The HWP1 is set at 45° to be the same condition as the setup in Fig.2. The HWP2 is set at -45° to reverse the polarization rotation by HWP1. The side view shows the height difference between the CW and CCW beams depending on the tilting angle of HWP1. A polarizer (POL) is set at 45° to make the output horizontal- and vertical-component of the beams interfere. The output beams are collected by a multimode fiber (M.M.F.) only, or the M.M.F. and 1 mm diameter irises, or a singlemode fiber (S.M.F.) only. The intensity of the laser output from the fiber is measured by an optical power meter.

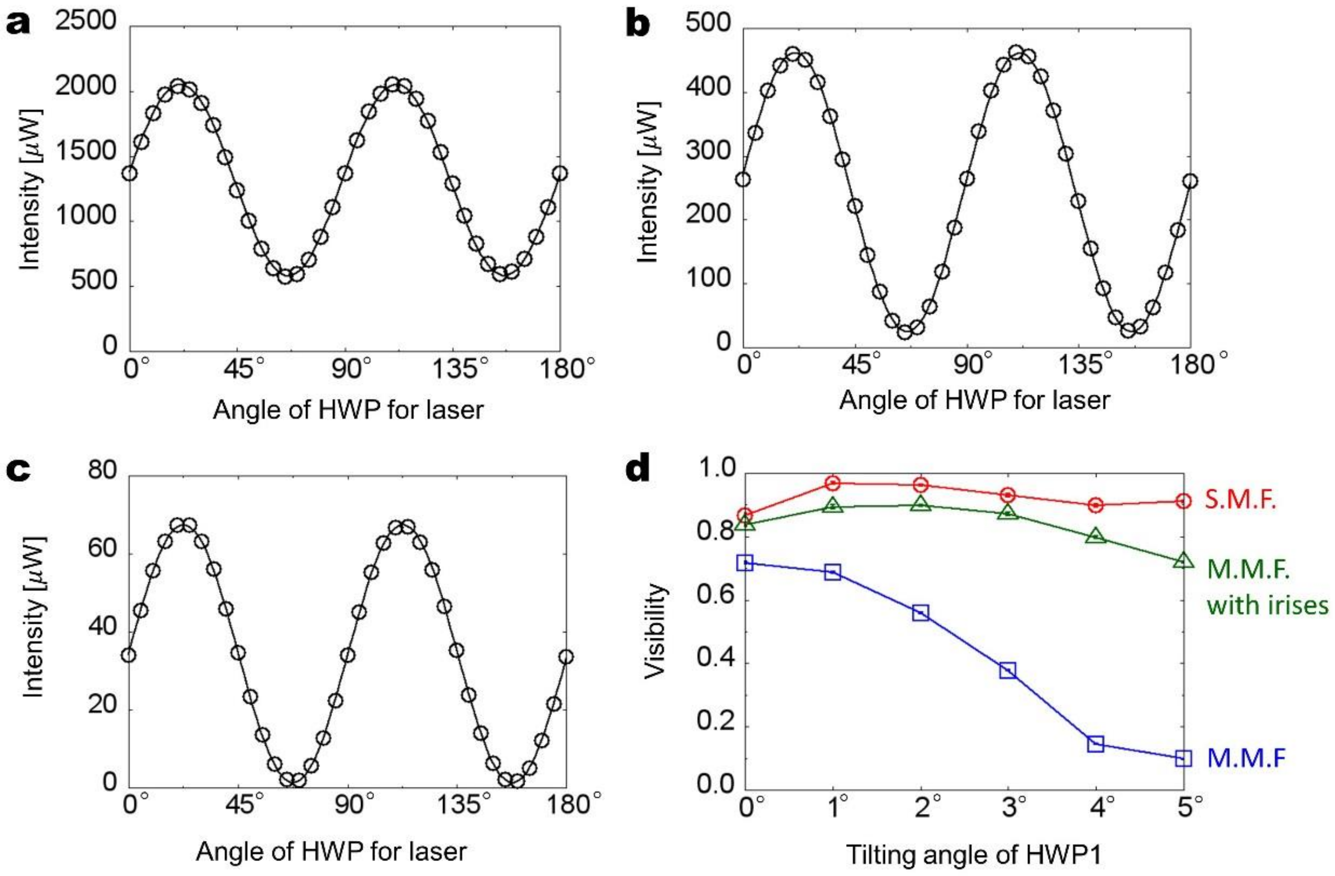

Supplementary Figure S2. Experimental results.

Plot of output intensity when the tilting angle of HWP1 is set at 2° as a function of the angles of HWP for laser using (a) a multi-mode fiber (M.M.F.) only, (b) the M.M.F. with 1 mm diameter irises, and (c) a singlemode fiber (S.M.F.) only. (d) Plot of measured visibilities as a function of the tilting angles of HWP1. Circle-, triangle-, and square-dots show the S.M.F. only case, the M.M.F. and irises case, and the M.M.F. only case, respectively.
